# Mitochondrial nanomotion measured by optical microscopy

**DOI:** 10.3389/fmicb.2023.1133773

**Published:** 2023-03-23

**Authors:** Priyanka Parmar, Maria Ines Villalba, Alexandre Seiji Horii Huber, Aleksandar Kalauzi, Dragana Bartolić, Ksenija Radotić, Ronnie Guy Willaert, Derrick F. MacFabe, Sandor Kasas

**Affiliations:** ^1^Laboratory of Biological Electron Microscopy, École Polytechnique Fédérale de Lausanne (EPFL) and University of Lausanne (UNIL), Lausanne, Switzerland; ^2^International Joint Research Group VUB-EPFL NanoBiotechnology and NanoMedicine (NANO), Vrije Universiteit Brussel and École Polytechnique Fédérale de Lausanne (EPFL), Lausanne, Switzerland; ^3^Institute for Multidisciplinary Research, University of Belgrade, Belgrade, Serbia; ^4^Alliance Research Group VUB-UGent NanoMicrobiology (NAMI), Research Group Structural Biology Brussels, Vrije Universiteit Brussel, Brussels, Belgium; ^5^Kilee Patchell-Evans Autism Research Group, London, ON, Canada; ^6^Department of Microbiology, Faculty of Medicine, Centre of Healthy Eating and Food Innovation (HEFI), Maastricht University, Maastricht, Netherlands; ^7^Centre Universitaire Romand de Médecine Légale, UFAM, University of Lausanne, Lausanne, Switzerland

**Keywords:** optical nanomotion, mitochondria, rotenone, metabolic substrates, short chain fatty acids

## Abstract

Nanometric scale size oscillations seem to be a fundamental feature of all living organisms on Earth. Their detection usually requires complex and very sensitive devices. However, some recent studies demonstrated that very simple optical microscopes and dedicated image processing software can also fulfill this task. This novel technique, termed as optical nanomotion detection (ONMD), was recently successfully used on yeast cells to conduct rapid antifungal sensitivity tests. In this study, we demonstrate that the ONMD method can monitor motile sub-cellular organelles, such as mitochondria. Here, mitochondrial isolates (from HEK 293 T and Jurkat cells) undergo predictable motility when viewed by ONMD and triggered by mitochondrial toxins, citric acid intermediates, and dietary and bacterial fermentation products (short-chain fatty acids) at various doses and durations. The technique has superior advantages compared to classical methods since it is rapid, possesses a single organelle sensitivity, and is label- and attachment-free.

## 1. Introduction

All living organisms seem to exhibit a nanometric scale motion referred to as nanomotion. These nanomotions last as long the organism is alive and ceases at cellular death. Although it is well-established that nanomotion is closely related to the metabolism of the living organism, its origin is not fully understood (Kohler et al., 2019). Cellular mechanisms such as cytoskeleton reorganizations, the opening of ionic channels, vesicle production and trafficking, single protein conformational changes, cellular pH as well as the activity of mitochondria have all been proposed as driving mechanisms inducing nanomotion. However, it is more likely that nanomotion results from the sum of these diverse biological processes rather than from a single one. Different techniques have been suggested to measure nanomotion (Longo et al., 2013; Syal et al., 2016; Rosłoń et al., 2022). The most popular technique is atomic force microscopy (AFM)-based nanomotion detection. In this method, a biological sample is attached to an AFM cantilever, and the nanometric scale displacements of the sample induce oscillations of the lever, which are detected by the AFM detector. By exposing the sample to different chemicals, such a setup can provide a fast and reliable viability assay. Indeed, such assays have been conducted on numerous living organisms, ranging from eukaryotic cells, such as mouse osteoblasts, human neurons (Kasas et al., 2015) and yeasts (Kohler et al., 2020), to prokaryotic cells such as mycobacteria (Mustazzolu et al., 2019) and *Bordetella pertussis* (Villalba et al., 2018). The ability to distinguish between live and dead organisms is imperative in the context of rapid antibiotic susceptibility testing (AST). Nanomotion-based AST allows determining bacterial response to antibiotic exposure in a few hours while conventional methods that depend on the growth rate of bacteria require at least 24 h or even weeks in the case of slowly growing microorganisms such as those involved in pertussis or tuberculosis in specific microbial facilities. Interestingly, the method not only works with cells but also with subcellular organelles. Stupar et al. (2017) demonstrated that isolated mitochondria from mammalian cells also exhibited nanomotions that induced cantilever oscillations and that these oscillations changed upon exposure to substrates and inhibitors.

Recently, an alternative method to AFM-based nanomotion detection was developed and successfully applied to yeast cells (Willaert et al., 2020). The novel method, referred herewith to as optical nanomotion detection (ONMD), uses a classical optical microscope equipped with a video camera to record the motions of the sample previously deposited on a glass surface. A dedicated software detects and measures the two-dimensional (2D) displacement of the cells. ONMD possesses several advantages if compared to the traditional AFM-based methods. It does not require the attachment of the cells to a substrate, it monitors single cells as well as the whole cellular population, is much simpler to set up, and is considerably more cost-effective.

To extend the ONMD application range, we monitored isolated mitochondrial nanomotion from established cell lines (HEK 293 T and Jurkat cells) with this new technique. Mitochondria, believed to originate from prokaryotic cells, live in eukaryotic cells in an endosymbiotic manner and are involved in numerous physiological processes throughout the lifecycle. They also play an underappreciated role in numerous pathological states such as diabetes, cardiac diseases, some cancers (Seyfried et al., 2014), long-haul Covid (Saleh et al., 2020), and several neurological disorders (MacFabe, 2015). Causes of mitochondrial dysfunction are complex, stemming from inherited mutations of mitochondrial or nuclear DNA, mitochondrial variation (heteroplasmy), but also diverse acquired factors including many environmental agents, drugs, metabolic intermediates, and even bacterial metabolites (MacFabe, 2015; Frye et al., 2016). These mechanisms are remarkably dose, time and tissue specific, need not be mutually exclusive, and may be reinforcing. Thus, elucidating the diverse causes of mitochondrial dysfunction is not an easy task. Among the available methods, we can mention the measurement of their ATP production, oxygen consumption, ion fluxes (i.e., electron, proton, and calcium gradients), lipid profiles, mitochondrial morphology, DNA extraction, and its sequencing. A simpler, faster, and cheaper alternative would be very welcome to democratize mitochondrial disease diagnostics and ONMD is a potential candidate for the task.

**Figure 1:**
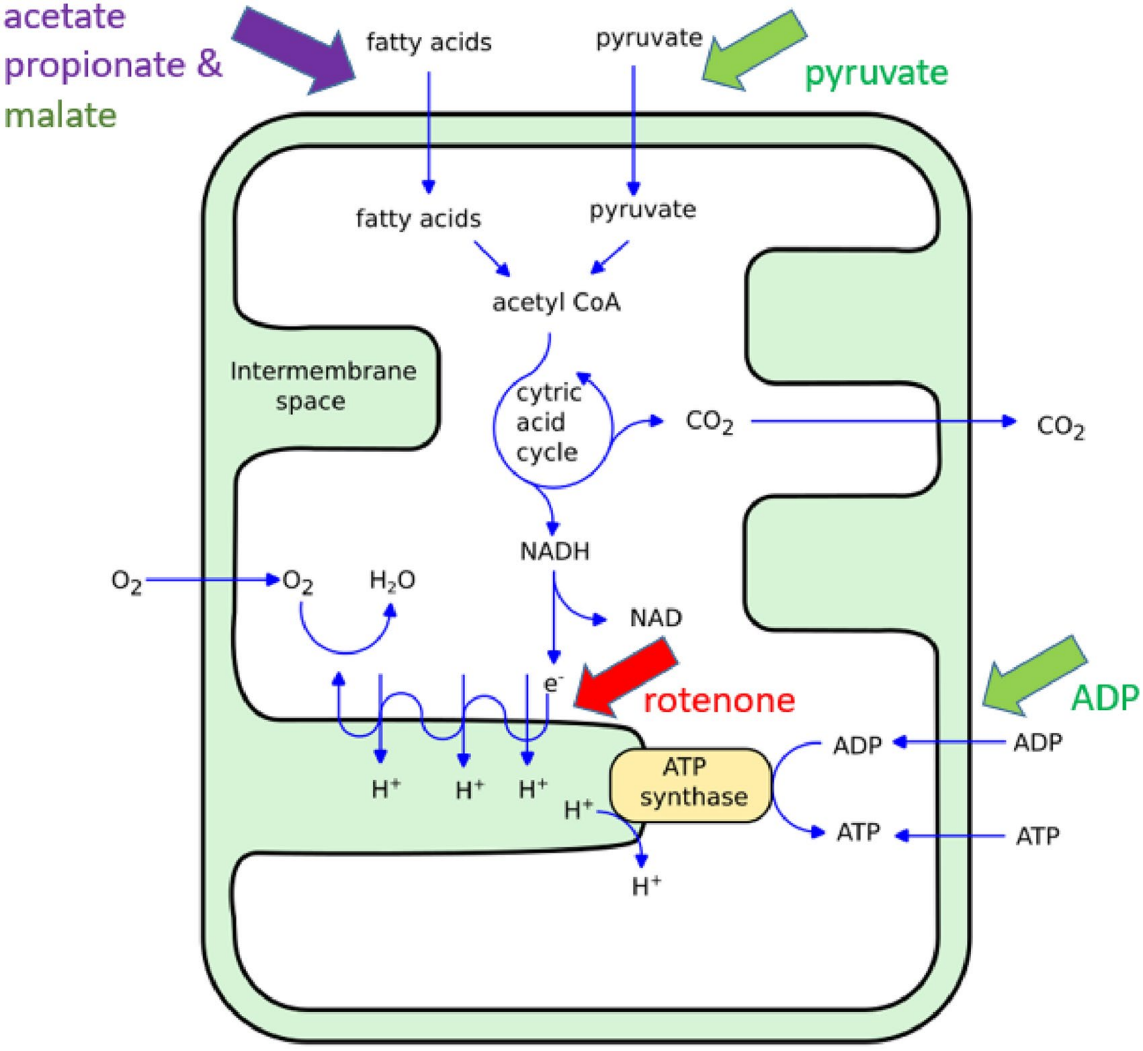
Illustration of the ATP synthesis in mitochondria. The chemicals used in this study induce an increase (green), a reduction (red) or more complex (violet) effects on mitochondria metabolism. For more detailed information on the specific effects of short chain fatty acids on mitochondrial function see (MacFabe, 2015; Frye et al., 2016).

To initially explore this potential, we conducted ONMD measurements of isolated mitochondria upon exposure to various established chemical stimuli such as rotenone and adenosine diphosphate (ADP; Figure 1). Rotenone is a specific inhibitor of the complex I of the mitochondrial electron transport chain (ETC; Sherer et al., 2003). The molecule is lipophilic, it can easily pass-through cell membranes and its accumulation in mitochondria blocks oxidative phosphorylation and causes a reduction of intracellular adenosine triphosphate (ATP) levels. Furthermore, respiratory chain dysfunction could lead to increased production of reactive oxygen species (ROS), which is a known factor to trigger apoptotic cell death (Siddiqui et al., 2013; Bavli et al., 2016). Stupar et al. (2017) demonstrated, using the AFM-based nanomotion detection method, that mitochondrial nanomotion was reduced upon rotenone exposure, and ADP added to malate and pyruvate stimulates mitochondrial respiration and increases mitochondrial nanomotion (Stupar et al., 2017).

In addition to these mitochondrial “inhibitors” and “activators,” we also conducted experiments involving the dietary and microbially produced short-chain fatty acids (SCFAs) such as propionate and acetate (Figure 1). These SCFAs, along with butyrate, have broad effects on cellular signaling, metabolism, immunity, and gene expression. Gut microbiota, following the anerobic fermentation of carbohydrates and some proteins, are the main producers of these molecules in humans. In addition, propionic acid is used as a common food preservative in processed grain and dairy products. It is produced by many disease-associated bacteria, while acetate is also endogenously produced (for comprehensive information see MacFabe, 2015). The SCFAs produced in the gut are rapidly absorbed by intestinal epithelial cells by passive and active means where, in addition to signaling, they provide host mitochondria with a major energy source *via* β-oxidation (Deleu et al., 2021), but also in high doses, metabolic dysfunction. Numerous studies have shown that SCFAs level imbalance may contribute to many chronic diseases such as diabetes, obesity (Adler et al., 2021), inflammatory bowel disease (Ormsby et al., 2020), and autism (MacFabe, 2015; Frye et al., 2016, 2017; Silva et al., 2020), through multiple processes including mitochondrial dysfunction. Of note, over the last 15 years, we have shown in animal models and human cell lines that SCFAs in high doses, in particular propionic acid, may inhibit mitochondrial TCA function through toxic intermediates, including nitropropionic acid and propionyl Co-A, or the sequestering of carnitine (MacFabe, 2015; Frye et al., 2016). Recently, we have also found that micromolar levels of SCFAs promoted the growth of human neural progenitor cells while millimolar levels had toxic effects (Yang et al., 2020). In this initial work, we exposed HEK239T cells isolated mitochondria to various doses and time exposures of propionate and acetate and monitored their nanomotion by ONMD.

In this proof of principle study, we validated the technique as an important tool in the exploration of the effect of mitochondrial toxins, citric acid intermediates, and common dietary and microbiome-produced molecules on mitochondrial physiology and toxicology. The results demonstrate that ONMD applied on isolated mitochondria is a promising tool to explore these organelle’s function, in a label- and attachment-free method.

## 2. Materials and methods

### 2.1. Cell culture

Jurkat cells (immortal cell line of human T cells; Simula and Campello, 2018) were cultured in RPMI (Gibco, 21875034) supplemented with 50 mL Fetal Bovine Serum (FCS; Gibco, 10270106) and 1% penicillin/streptomycin (100 Units/mL; Gibco, 15140122) in a 5% CO_2_ incubator set to 37°C. Cells were split 1:10 every 3–4 days. Human embryonic kidney cells (HEK293T; Lampl et al., 2015), were cultured in Dulbecco’s modified Eagle medium (DMEM; Gibco, 31,966,021) supplemented with 50 mL of FCS and 1% penicillin/streptomycin (100 Units/mL) in a 5% CO_2_ incubator set to 37°C. Cells were split to 1:3 when confluency reached 80% (every 3–4 days). The cell number was determined manually by using a Neubauer hemocytometer.

### 2.2. Mitochondrial isolation

**Figure 2:**
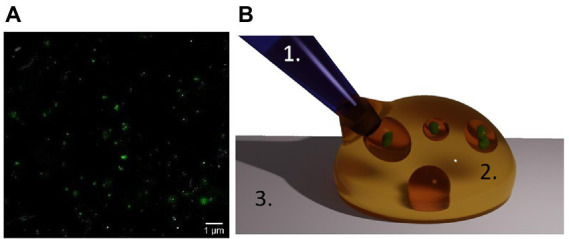
Isolated mitochondria and oil encapsulation. **(A)** Mitochondria as seen by optical fluorescent microscopy after SYBR green staining of the mt-DNA. **(B)** Suspension of mitochondria in water-in-oil droplets in our assay. A solution containing mitochondria (green) in their culture medium (plus the chemical which action on the organelles is under study), is manually injected using a micro-pipette (1) into an oil droplet (2) on a glass surface (3).

Mitochondria were isolated from HEK293T and Jurkat cells using the Qproteome® Mitochondria Isolation Kit (Qiagen, Cat. # 37612) following the manufacturer’s protocol. Before the isolation, cells (between 4 × 10^6^ and 1 × 10^7^ cells) were collected in a 15 mL conical tube by centrifugation at 500 × g for 10 min at 4°C. The pellet was resuspended in 1 mL of 0.9% w/v sodium chloride solution before pelleting again by centrifugation at 500 × g for 10 min at 4°C. Washed cells were used to isolate mitochondria following the isolation kit protocol. Lastly, the final mitochondrial pellet was suspended in Mitochondria Storage buffer (MSB) from the isolation kit and stored at 4°C until further use. All the differential centrifugation steps were conducted at 4°C using a high-speed refrigerated micro centrifuge (Himac CT 15RE). In only one case, the mitochondria suspension was incubated for 15 min with SYBR green to stain the mitochondrial DNA (mt-DNA) and to confirm the success of the isolation process (Figure 2). To visualize the isolated mitochondria solution, AFM topographic images were taken (Supplementary Figure S1).

### 2.3. Rotenone treatment

Isolated mitochondria from HEK293T and Jurkat cell lines were either untreated (served as a control) or treated for 40 min with 1 μM rotenone (Sigma, R8875), which is an established inhibitor of mitochondrial electron transport (Frye et al., 2016). After the incubation, the rotenone was removed by centrifugation at 6,500 g for 5 min and the pellet was resuspended in 10 μL MSB. The rotenone concentration and exposure time treatment were selected for consistency with previous reports (Stupar et al., 2017).

### 2.4. Metabolic substrates treatment

Isolated mitochondria from HEK293T cells in MSB buffer (from the kit) were incubated for 15 min with substrates involved in the citric acid cycle. Malate (AppliChem, A7947) and pyruvate (Sigma, S8636) at 5 mM concentration, while ADP (Sigma, A2754) was added in a concentration of 1 mM. Rotenone at 1 μM concentration was finally added as nanomotion inhibitor. Consistency with previous reports was considered when selecting the concentration and exposure time treatments (Stupar et al., 2017).

### 2.5. Propionate treatment

Mitochondria isolated from HEK293T cells were either untreated (served as a control) or treated with five concentrations of sodium propionate (Sigma, P1880; 2 μM, 200 μM, 800 μM, 3.2 mM, 12.8 mM) based on our previous studies (Yang et al., 2020) for 2 h and overnight.

### 2.6. Acetate treatment

Mitochondria isolated from HEK293T cells were either untreated (served as a control) or treated with five concentrations of sodium acetate trihydrate (Alfa Aesar, 11553; 30 μM, 3 mM, 12 mM, 48 mM, 192 mM; Yang et al., 2020) for 2 h and overnight.

### 2.7. Encapsulation of isolated mitochondria in water-in-oil droplets

To minimize the adhesion of mitochondria to the walls of the analysis chambers the organelles were isolated in water-in-oil droplets (see Figure 2). One drop of immersion oil [InmersolTM 518F oil (Zeiss)] or HFE 7500 fluorinated oil (3MTM Novec™) with the surfactant Pico-Surf™ (2% w/w in Novec™ 7500) was deposited on a glass microscopy slide. Then, 5–20 μL of freshly isolated mitochondria were gently added into the oil drop using a pipette. This resulted in water-in-oil droplets of varying sizes containing many isolated mitochondria. Subsequently, the droplets were extracted from the water-in-oil emulsion with a pipette and deposited into the analysis chambers.

### 2.8. Custom-made microfluidic device manufacturing

**Figure 3:**
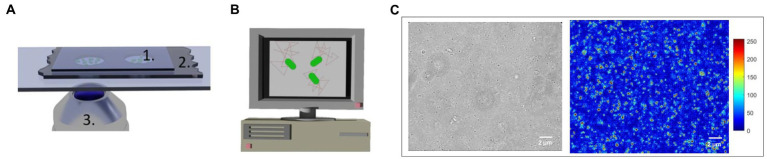
Image acquisition set-up (not to scale) **(A)** 1. Analysis chamber with mitochondria 2. Double-sided 10 μm thick adhesive foil, 3. Optical microscope objective. **(B)** Computer screen displaying mitochondrial displacements. **(C)** Optical and changing pixel typical images of a group of mitochondria undergoing nanomotion. The false colors code for the nanomotion amplitude in arbitrary units (blue: immobile, red: highly motile organelles).

Custom-made microfluidic devices were prepared by cutting ~4.5 cm × 2 cm rectangles from a PET-double-sided A4 foil (10 μm thick; N°. Nitto 5601-L, color: transparent, Permapack). Eventually two central, circular holes were punched in it by using a puncher. The bottom layer of the double-sided foil was peeled off and the foil was stuck onto a microscope sample holder glass. About 0.5 μL of the solution containing the mitochondria were deposited in the analysis chambers. Finally, the upper layer of the double-sided foil was peeled off using forceps and a 24-mm-glass coverslip sealed the chambers (Figure 3A).

### 2.9. Displacement data acquisition

An inverted optical microscope (Zeiss Observer Z.1) with a 63 × oil-immersion objective, coupled to a camera (MU9PC-MH XIMEA and PCO Edge 5.5) was used to record the 2D displacement of single mitochondria. The camera was typically recording 10-s-long videos with a framerate of 30 frames per second (fps). The recorded movies were saved as AVI files. All measurements were carried out at room temperature without phase contrast nor fluorescent staining of the mt-DNA.

A custom-made tracking algorithm implemented in MATLAB tracked the 2D displacements of manually selected mitochondria. The algorithm calculated the organelles total displacements and their velocity distribution over 120 and 200 frames (Figure 3). A more detailed description of the analysis method can be found elsewhere (Willaert et al., 2020).

### 2.10. AFM-based nanomotion

Mitochondria isolated from HEK293T were attached to Phenotech Sensors (Resistell AG, RA01) using Functionalization Buffer A (Resistell AG, GNBxxx^1^-01) and Washing Buffer (Resistell AG, GNBxxx^1^-02) from the Phenotech sample preparation kit—Gram-negative bacteria (Resistell AG, GNR050-00), according to the manufacturer’s protocol. The measurements were done using Phenotech Research device (Resistell AG). The sensors with and without mitochondria attached were immersed during the measurements in Mitochondria Storage buffer (QIAGEN).

### 2.11. Signal frequency analysis

The mean power spectral density (PSD) profiles for mitochondria ONMD signals were obtained using MATLAB pwelch command in the X and Y direction (window length of 169 samples; overlapping 20 samples). All PSD profiles of 10 signals referring to control, as well as 25 signals derived from rotenone-treated samples were averaged. PSD profiles for AFM-based nanomotions with/without mitochondria were also obtained using the pwelch command in MATLAB (size window: 80 samples, overlapping 20 samples). It was observed that in the total frequency range from 0 to 250 Hz, all activity was only located up to 10–15 Hz. Therefore, we displayed the spectra up to 15 Hz only, to be analogous to the ONMD registration.

### 2.12. Statistical analysis

We used OriginLab 2018b for graphing and statistical analysis. The results were expressed as the mean ± SEM of at least 3 replicates of independent experiments. Differences among treatments were determined using Welch’s ANOVA, followed by Games-Howell’s multiple comparisons test. *p*-Values below 0.05 were considered significant. Welch’s ANOVA is an alternative to the traditional analysis of variance (ANOVA), and it is used when the variances and sample sizes are unequal.

## 3. Results

### 3.1. Effect of rotenone on mitochondrial nanomotion

**Figure 4:**
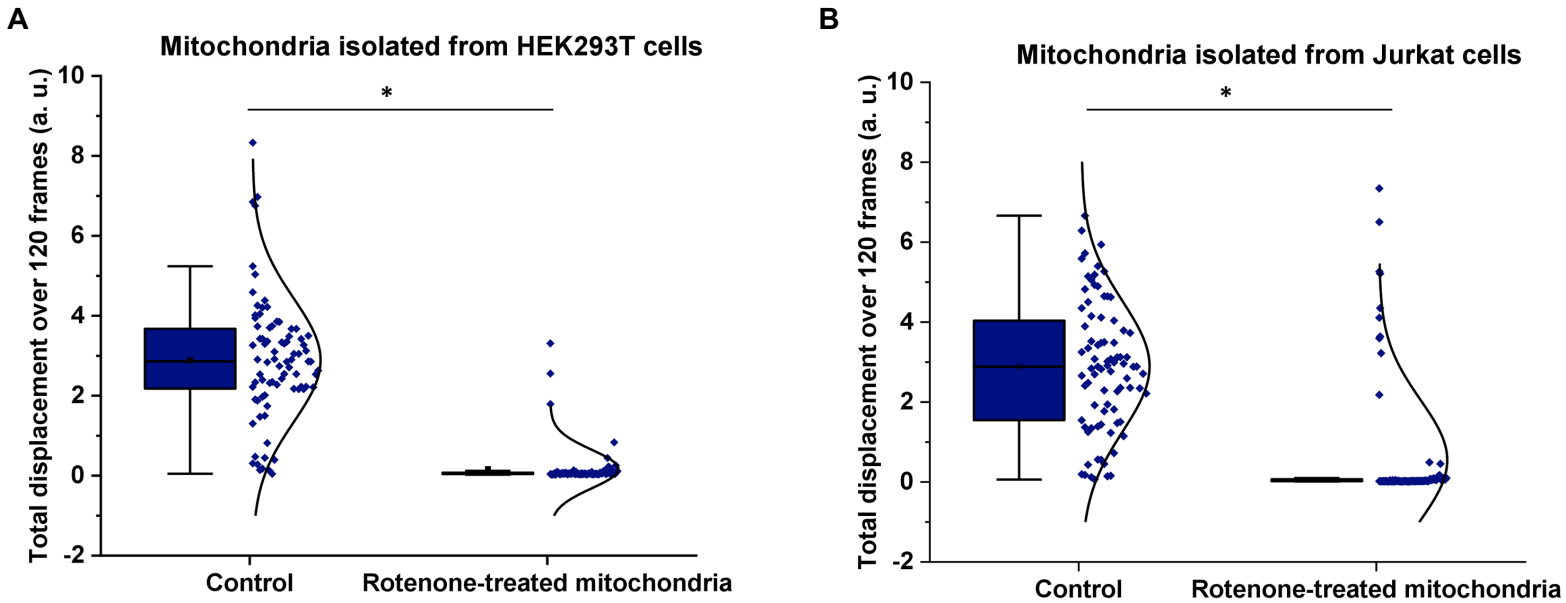
Effect of rotenone on mitochondrial nanomotion. Mitochondria were either untreated or exposed to 1 μM rotenone for 40 min. Mitochondrial displacement values are expressed in arbitrary unit (a.u.). **(A)** Effect of rotenone on the 2D displacement of mitochondria isolated from HEK293T cells. In the control treatment, the displacement of 82 single mitochondria were measured whereas 85 in the case of rotenone treatment. **(B)** Effect of rotenone on the 2D displacement of mitochondria isolated from Jurkat cells. In the control treatment, 83 different mitochondria were measured whereas 90 in the case of rotenone treatment. Results are representative of three independent experiments. *p < 0.005 from two-sample *t*-test.

In this set of experiments, we used the ONMD method to examine the effect of rotenone on the nanomotion of mitochondria isolated from HEK293T cells. As mentioned previously, rotenone is a specific inhibitor of the complex I of the mitochondrial electron transport chain (ETC), *via* the inhibition of ubiquinone reductase (Frye et al., 2016; Figure 1). Freshly isolated mitochondria were either untreated (served as the control) or treated with 1 μM rotenone for 40 min. As shown in Figure 4A, treatment with rotenone led to a significant reduction of the mitochondrial nanomotion, which was consistent with its toxic effect and similarly occurred after fixation with glutaraldehyde (GA; Supplementary Figure S2). A non-toxic volume of DMSO (as an inert control) was tested on mitochondrial nanomotion and no significant differences were observed (Supplementary Figure S3).

To further confirm that mitochondrial nanomotion was dramatically affected by rotenone, the same rotenone treatment was applied to mitochondria isolated from Jurkat cells (Figure 4B). The results were consistent with those obtained with mitochondria isolated from HEK239 cells. These experiments confirm earlier findings (Stupar et al., 2017) that rotenone inhibits mitochondrial activity, which results in the arrest of mitochondrial measurable nanomotion in our assay.

### 3.2. Effect of ADP, malate, and pyruvate on mitochondrial nanomotion

**Figure 5:**
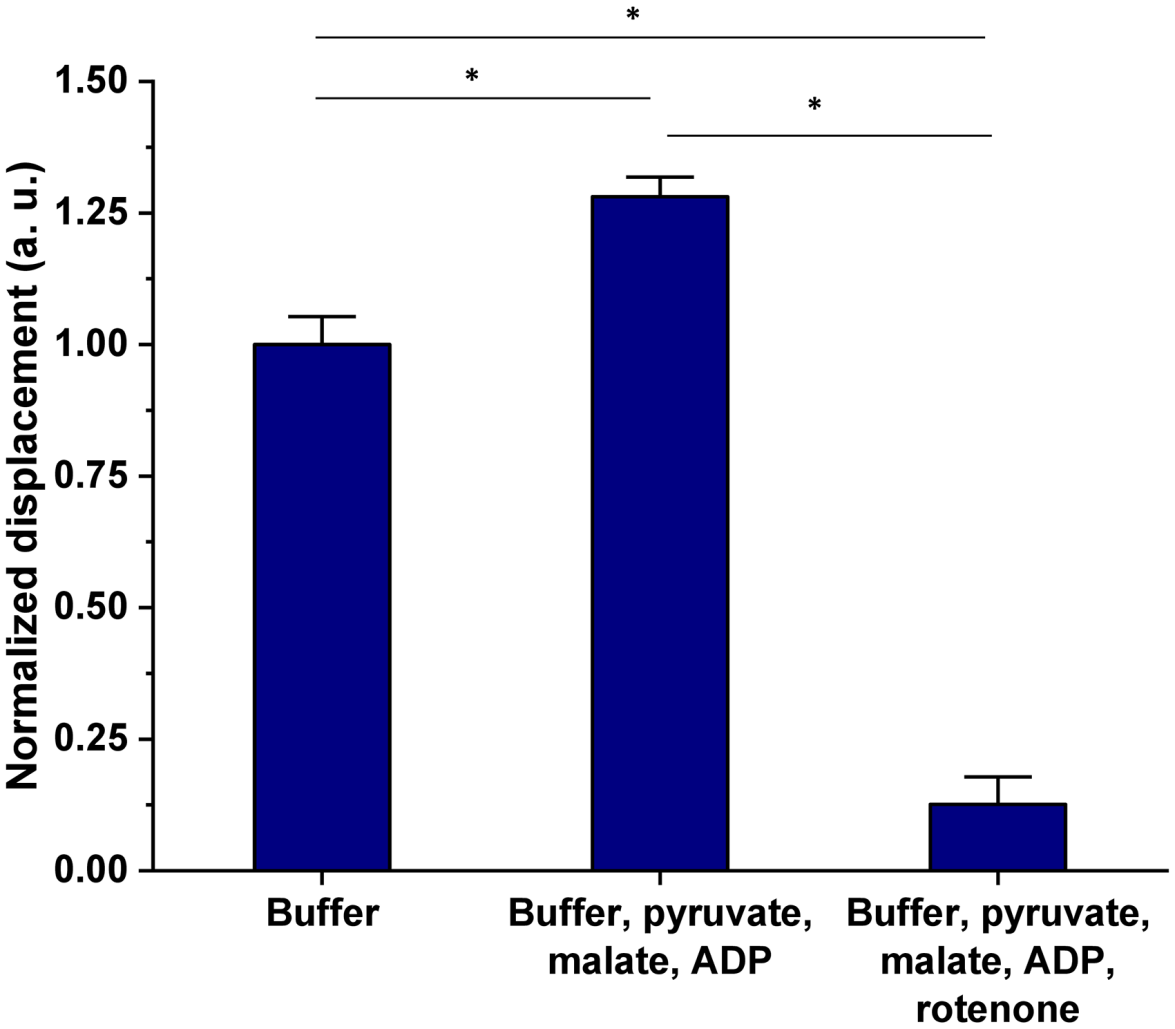
Effect of 15 min exposure of mitochondria isolated from HEK293T cells, to pyruvate, malate, and ADP on mitochondrial nanomotion as compared to control and “dead” (rotenone treated) organelles. Results are presented as mean ± SEM and are representative of three independent experiments. The number of mitochondria analyzed in each treatment was as follows: buffer = 237, buffer + 5 mM pyruvate + 5 mM malate + 1 mM ADP = 339, buffer + 5 mM pyruvate + 5 mM malate + 1 mM ADP + 1 μM rotenone = 83. *p value <0.005.

Freshly isolated mitochondria from HEK293T cells were incubated in MSB buffer complemented with 3 key citric acid cycle (Krebs) substrates, i.e., pyruvate, malate, and ADP (Figure 1). After 15 min of incubation, the suspension was introduced in the analysis chamber and the organelles’ nanomotion recorded. The latest increased by about 25% in the buffer supplemented with pyruvate, malate, and ADP as compared to the control sample (see Figure 5). These results clearly indicate a stimulation of the organelles by their substrates. Nanomotion of mitochondria incubated with the substrates and rotenone dramatically dropped the mitochondrial activity. The results are normalized to the buffer measurement (control) and represent the average displacement in each experimental condition.

### 3.3. Effect of propionate on mitochondrial nanomotion

**Figure 6:**
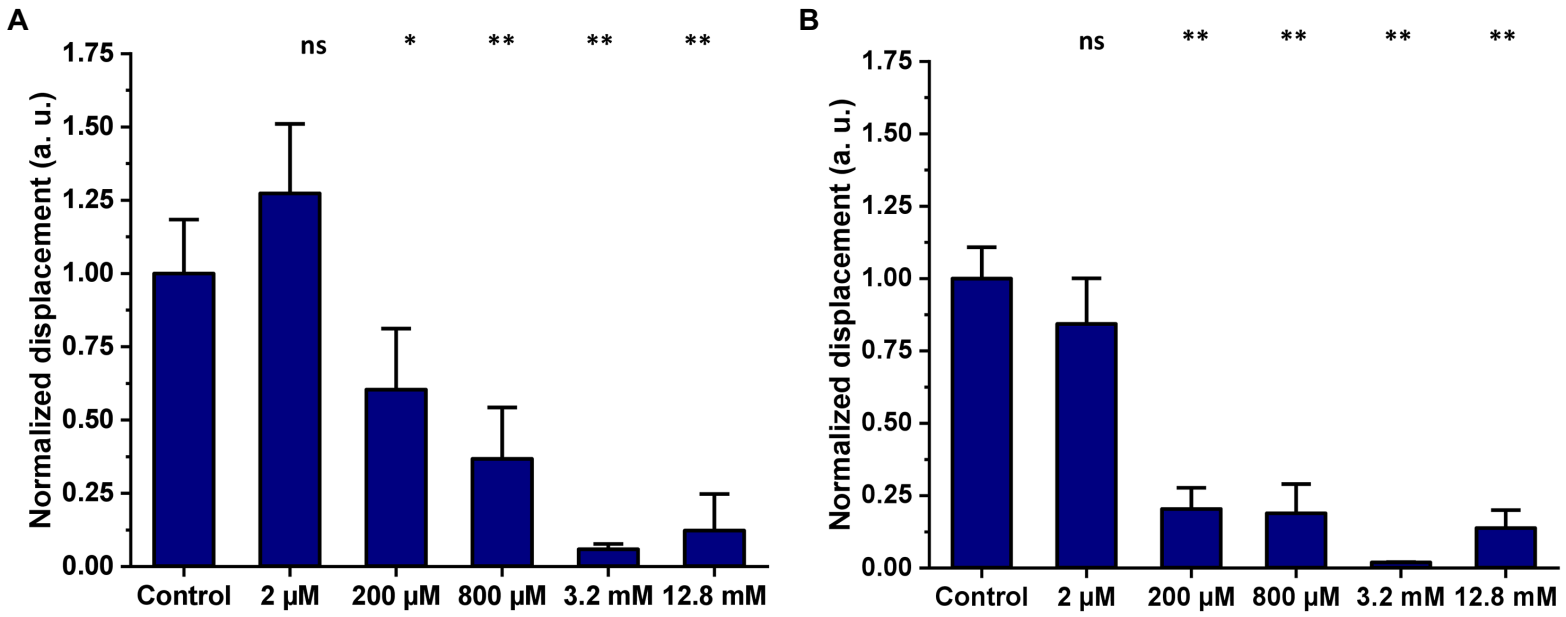
Mitochondria isolated from HEK293T were either untreated (control) or exposed to five different concentrations of propionate for 2 h and overnight incubation. Mitochondrial displacement values are expressed in arbitrary units (a. u.) and data are presented as mean ± SEM. Graphs are annotated with *p*-values from Welch’s ANOVA, followed by Games-Howell’s multiple comparisons test; ^*^p-value < 0.05, ^**^p < 0.001. Results are representative of three independent experiments. **(A)** Effect of increasing concentrations of propionate on mitochondrial displacement after a 2-h exposure. The number of mitochondria analyzed in total for each treatment was as follows: control 2 h = 39, 2 μM = 38, 200 μM = 31, 800 μM = 38, 3.2 mM = 55, 12.8 mM = 48. **(B)** Effect of increasing concentrations of propionate on mitochondrial displacement after overnight exposure. The number of single mitochondria analyzed in total for each treatment was as follows: control overnight = 63, 2 μM = 47, 200 μM = 65, 800 μM = 60, 3.2 mM = 64, 12.8 mM = 58.

Mitochondria were isolated from HEK239T cells and subsequently exposed to propionate for 2 h and overnight. The same five concentrations of propionate (2 μM, 200 μM, 800 μM, 3.2 mM, and 12.8 mM) used in our previous study (Yang et al., 2020) were applied. The 2D displacements of single mitochondria treated with propionate were measured after a 2-h and after overnight incubation and were compared to the untreated control. Figure 6 shows the effect of increasing propionate concentrations and exposure time on the nanomotion of isolated mitochondria. Exposure of isolated mitochondria to propionate for 2 h showed a significant reduction in mitochondrial displacement at 3.2 and 12.8 mM over untreated control (Figure 6A), and this effect was still observable after the overnight incubation (Figure 6B).

### 3.4. Effect of acetate on mitochondrial nanomotion

**Figure 7:**
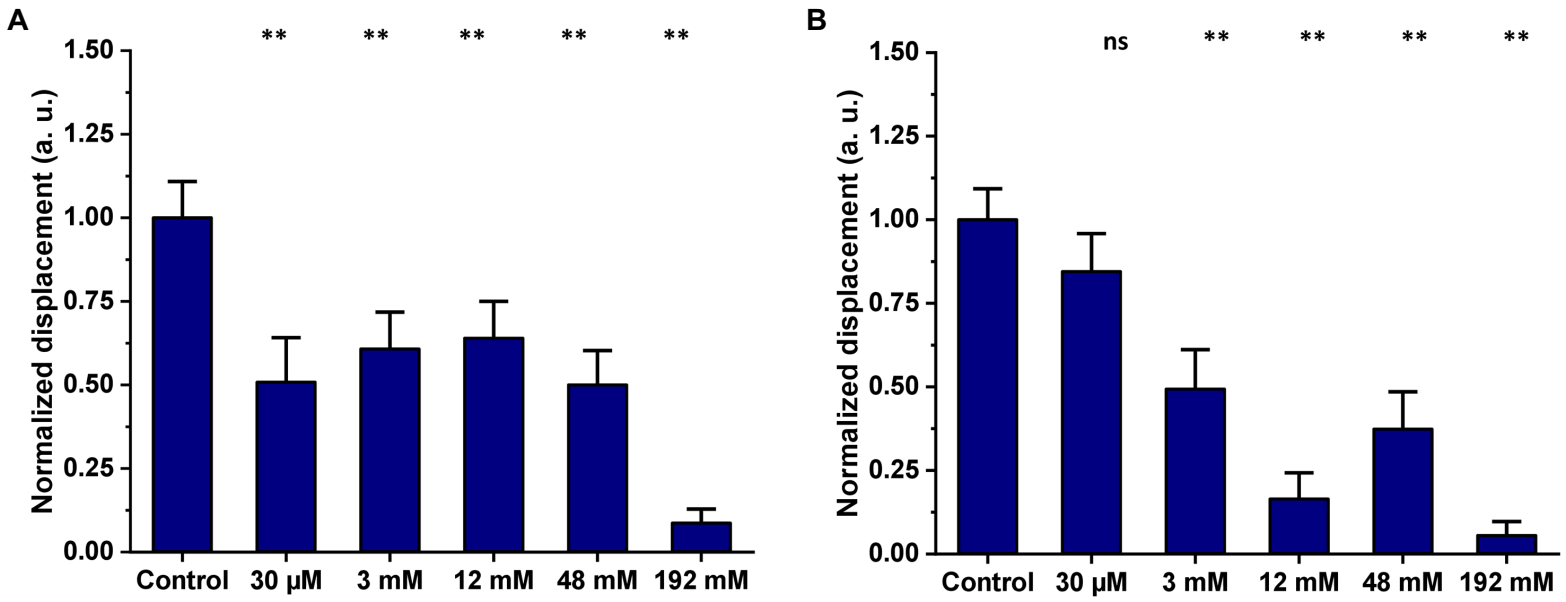
Effect of increasing concentrations of acetate on mitochondrial nanomotion after **(A)** a 2-h exposure and **(B)** overnight exposure (mitochondria isolated from HEK293T cells). Results are representative of three independent experiments. Graphs are annotated with *p*-values from Welch’s ANOVA, followed by Games-Howell’s multiple comparisons test; ^**^p < 0.001. The number of single mitochondria analyzed after 2 h incubation was: control = 55, 30 μM = 32, 2 mM = 51, 12 mM = 50, 48 mM = 61, 129 mM = 49. The number of single mitochondria analyzed after overnight incubation was: control overnight = 43, 30 μM = 41, 2 mM = 42, 12 mM = 40, 48 mM = 42, 129 mM = 40.

HEK293T cell mitochondria were either untreated or exposed to five different concentrations of acetate for 2-h and overnight incubation. The mean total displacement of single mitochondria treated with acetate was calculated and compared to the untreated control. In Figure 7, mitochondrial displacement values are expressed in arbitrary units (a.u.) and data are presented as mean ± SEM.

### 3.5. Signal frequency of mitochondria acquired by ONMD and AFM-based nanomotion

**Figure 8:**
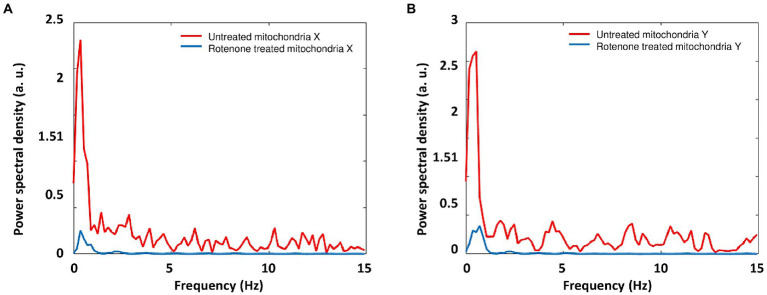
Mean PSD profiles for mitochondria ONMD signals in the X (**A**) and Y direction (**B**). Red: untreated mitochondria; Blue: rotenone treated mitochondria.

The power spectral density (PSD) profiles for mitochondria ONMD signals were calculated (Figure 8). It can be seen from Figure 8 that rotenone inhibited mitochondrial motion in all frequencies of the spectral range (0.1775–15 Hz; initial frequency 30/169 = 1/5.633 = 0.1775 Hz; highest frequency 30/2 = 15 Hz). Although the most active range is spread up to approx. 1.5–2 Hz; we can observe that other spectral components were also inhibited; probably originating from active mitochondrial motion. A more precise upper limit of that biological motion frequency range can be obtained by increasing the number of frames/s.

**Figure 9:**
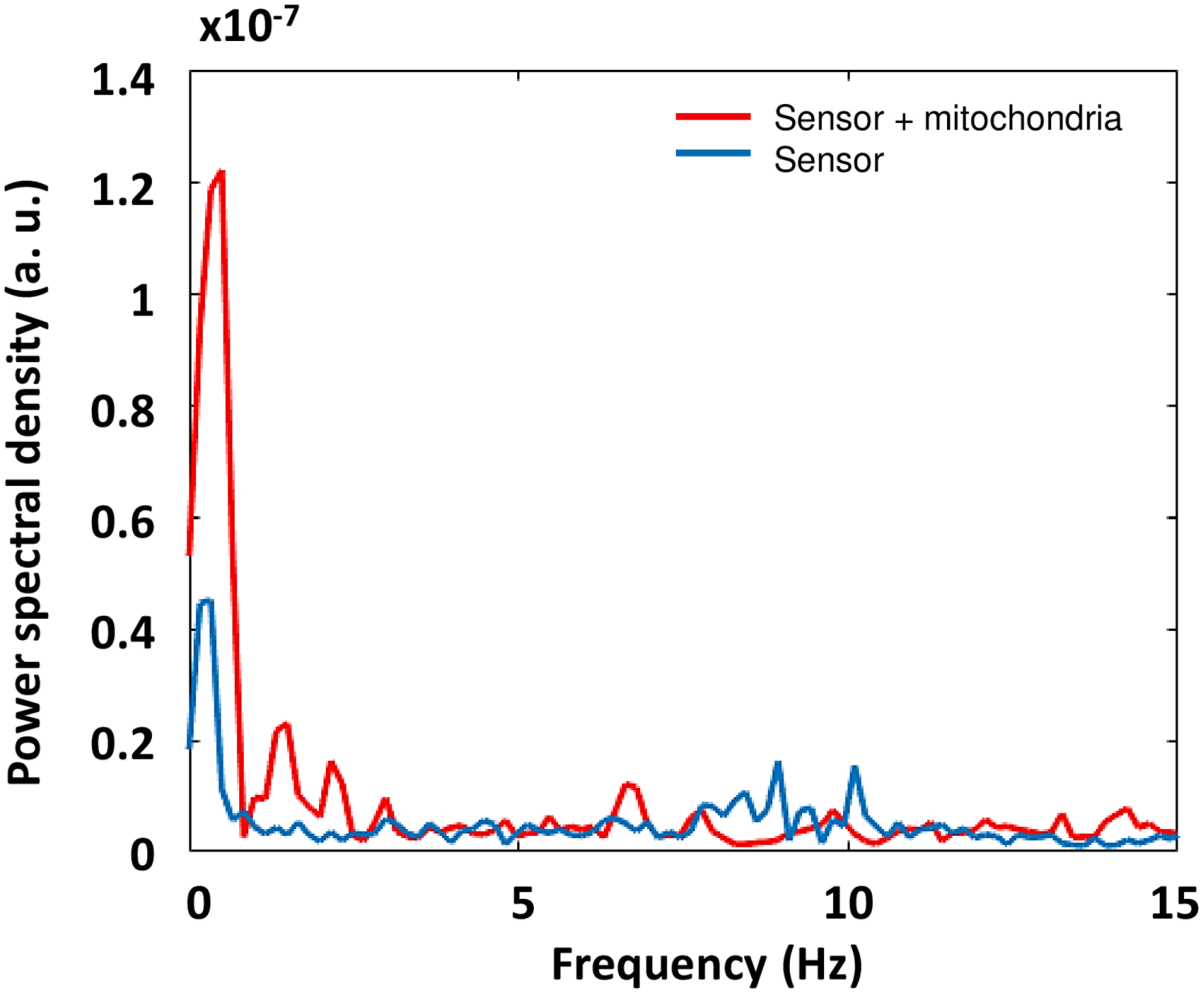
PSD profiles of AFM-based mitochondrial nanomotion for sensor without (blue) or with mitochondria (red) attached, with the frequency axis limited to 15 Hz.

When observing AFM-based nanomotion signal spectra in Figure 9, it shows that mitochondrial nanomotions contributed mostly to the activity in the low-frequency region (0.1655 – approx. 2 or 3 Hz). The initial frequency was obtained as the reciprocal value of the signal duration 500/3021 = 1/6.0420 = 0.1655 Hz. Although empty sensors displayed some activity in the lowest region (up to approx. 1 Hz; Figure 9: blue line), mitochondria added significantly to this activity (Figure 9: red spectrum). Interestingly, mitochondria also showed some activity in the next region (approx. 1–3 Hz), where empty sensors showed no activity.

As conclusion, when comparing mitochondrial activity recorded with ONMD and AFM (Figures 8, 9), we can observe that with the ONMD registration, mitochondrial activity, although most intensive in the first few Hz, also existed in the region up to 15 Hz (maybe at even higher frequencies, but obtainable only with a greater number of frames/s). This conclusion was derived from the fact that rotenone inhibited mitochondrial activity in the whole observed frequency range (Figure 8). In the case of AFM registration, mitochondria also displayed the most pronounced activity in the lowest frequency range (Figure 9).

## 4. Discussion

In this study, we demonstrated that the ONMD technique can be successfully applied to measure the nanomotion of mitochondria in a predictable manner *via* the application of key pharmacological agents from diverse sources, including defined mitochondrial inhibitors and TCA intermediates, but also microbiome fermentation products. The variety of mitochondrial treatments and their specific durations were selected based on previous research (Stupar et al., 2017; Yang et al., 2020). In the first set of experiments, we used the ONMD technique to evaluate the effect of rotenone on nanomotion of mitochondria isolated from an established cell line—i.e. HEK239T cells—used in our laboratory but also in the study of mitochondrial function (Lampl et al., 2015; Simula and Campello, 2018). We showed that the addition of 1 μM rotenone to freshly isolated mitochondria led to the complete loss of mitochondrial displacement activity. This loss of activity is most likely due to the toxicity of rotenone as reported in numerous studies (Giordano et al., 2012; Siddiqui et al., 2013; Bavli et al., 2016; Frye et al., 2016; Carli et al., 2021). In addition, our results are in line with a previous study using the AFM-based nanomotion method (Stupar et al., 2017). The nanomotion PSD obtained by both methods were similar. The incubation of mitochondria with metabolic substrates (pyruvate, malate, and ADP) caused the nanomotion of the mitochondria to increase. The addition of a diphosphate used for conformational changes that precede the ATP synthesis amplified the displacements. The nanomotions that we observed with isolated mitochondria were highly dependent on the chemicals to which they were exposed. and they are similar to those of bacteria and yeast cells (Supplementary Figure S4).

Next, we used the ONMD technique to explore the effect of propionate and acetate, two dietary and microbiome-produced SCFAs with both beneficial and potentially harmful biological effects on mitochondrial nanomotion. The toxic effects of these molecules can have implications for many disease processes, including our established animal/*in vitro* model of autism (for comprehensive information, see MacFabe, 2015). The concentrations of SCFAs applied in this study were in the micromolar and millimolar ranges. We found that millimolar levels of propionate (3.2–12.8 mM) and acetate (3–192 mM) negatively impacted the displacement of isolated mitochondria after a 24-h exposure. These results are like those of previous studies that reported that only millimolar levels of SCFAs, including propionate and acetate, increased the apoptosis rate in human neural progenitor cells after 24 h (Yang et al., 2020), and in low doses facilitated, but at high doses inhibited, mitochondrial TCA cycle function in cultured lymphoblasts isolated from neurotypical children and those with autism spectrum disorder (Frye et al., 2016). However, we discovered in this study that micromolar levels of propionate (200 and 800 μm) also reduced the mitochondrial nanomotion. Overall, the experiments carried out in the first part of this work are consistent with our hypothesis that mitochondria exhibit nanomotion behavior that is dependent on their metabolic state.

Limitations and future directions: At this preliminary stage, it must be noted that the effects of these microbiome products are extremely complex and include cell signaling, intracellular pH, redox, membrane lipid profiles, and gene expression which may act alone or synergistically with the above processes to affect mitochondrial function. Some of the above may not be relevant as we used mitochondrial isolates from “naive” cells. Of note, these cell cultures were derived from malignant cell lines, which may have abnormal mitochondria (Seyfried et al., 2014). We do not yet know whether these effects are reversible or enduring. As well, ranges in mitochondrial function in our assay may be a function of experimental error, selection for “resistant” mitochondria during purification, or varying effects of these agents on subpopulations of mitochondria secondary to mitochondrial heteroplasmy. As we used mitochondrial isolates from untreated, intact cell cultures, the effect of these agents on mitochondrial function when exposed to whole cells of various types, or whole organisms as in our previous studies (MacFabe, 2015; Frye et al., 2016) are not yet known. These await future experimentation and confirmation. However, we have recently shown that systemic exposure to propionate in rats has shown measurable behavioral but also ultrastructural changes in hippocampal synaptic mitochondria *via* EM microscopy, consistent with our hypothesis (Lobzhanidze et al., 2020). Future studies will be directed at comparing different known treatments that affect different aspects of mitochondrial function. In summary, we report that optical nanomotion detection (ONMD) is a useful rapid and single mitochondria assay to examine and quantify the effect of diverse agents, including dietary and microbiome-produced metabolites on acquired mitochondrial function and dysfunction, an emerging factor in many chronic health conditions.

## Data availability statement

The original contributions presented in the study are included in the article/Supplementary material, further inquiries can be directed to the corresponding authors.

## Author contributions

SK, DM, RW, and MV conceived the study. SK, RW, and DM supervised the work. PP, AH, and MV cultivated the cells, isolated the mitochondria and conducted the ONMD measurement. AK, DB, KR analyze ONMD and AFM-based nanomotion signals frequencies. AK, DB, KR, PP, MV, RW, DM, and SK wrote the draft manuscript. All the authors contributed to the article and approved the submitted version.

## Funding

This research was funded by the Belgian Federal Science Policy Office (Belspo) and the European Space Agency (ESA), grant number PRODEX project Flumias Nanomotion; FWO, grant numbers I002620; and FWO-SNSF, grant number 310030L_197946. MV and SK were supported by SNSF grants CRSII5_173863. AK, DB, and KR were supporting by the Ministry of Science, Technological Development and Education grant: 451-03-47/2023-01/20005.

## Conflict of interest

The authors declare that the research was conducted in the absence of any commercial or financial relationships that could be construed as a potential conflict of interest.

## Publisher’s note

All claims expressed in this article are solely those of the authors and do not necessarily represent those of their affiliated organizations, or those of the publisher, the editors and the reviewers. Any product that may be evaluated in this article, or claim that may be made by its manufacturer, is not guaranteed or endorsed by the publisher.
