# Supplementary material for: Mitochondrial nanomotion measured by optical microscopy
Source: Front Microbiol. 2023 Mar 23;14:1133773. doi: 10.3389/fmicb.2023.1133773 (PMC10078959; doi:10.3389/fmicb.2023.1133773)
Supplement: Supplementary file 1 [file Data_Sheet_1.docx]

Supplementary Material

Mitochondrial nanomotion measured by optical microscopy

Priyanka Parmar, Maria I. Villalba, Alexandre Seiji Horii-Huber, Aleksandar Kalauzi, Dragana Bartolić, Ksenija Radotić, Ronnie G. Willaert, Derrick F. MacFabe, Sandor Kasas

# Supplementary Data

## Sample preparation and AFM measurement

An isolated mitochondria solution was attached to mica using 0.5% glutaraldehyde. The mica with mitochondria attached was covered with a drop of 0.5% glutaraldehyde for final fixation of the sample. AFM topographic and amplitude images were taken using a NX10 AFM (Park systems). Images were collected using PPP-NCHR 10 M cantilevers (Nanosensors, Switzerland) with a spring constant of 42 N/m. SmartScan operating software (v. 1.0.6, Park systems) has been used for the data acquisition and XEI software (v. 1.8.4, Park systems) for processing the images.

## DMSO treatment

Isolated mitochondria from HEK293 cells were incubated in mitochondria storage buffer (QIAGEN) with and without 0.125% v/v dimethyl sulfoxide (DMSO) (Sigma, D5879). The mitochondria suspensions were placed in custom-made microfluidic devices and the displacement data acquisition and analysis were performed as mentioned in the manuscript.

## Bacteria and yeast growth conditions

*Escherichia coli* (DH5α) single colonies were growth in Luria-Bertani (LB) (Invitrogen, 12780052) broth overnight at 37°C with shaking at 160 revolutions per minute (rpm). *Candida albicans* colonies were growth in yeast-extract peptone dextrose (YPD) medium (D-glucose: Sigma, G7528; yeast extract: Fluka, 09182; peptone: Sigma, 82303) overnight at 30°C and 160 rpm. The cultures were centrifugated and 1:7 diluted in fresh LB or YPD, depending the cell. The bacteria and yeast suspensions were deposited in the custom-made microfluidic devices and the displacement data acquisition and analysis were done as described in the manuscript. The *C. albicans* isolate 101 was kindly provided by Salomé Leibundgut and Christophe D’Enfert laboratories.

# Supplementary Figures


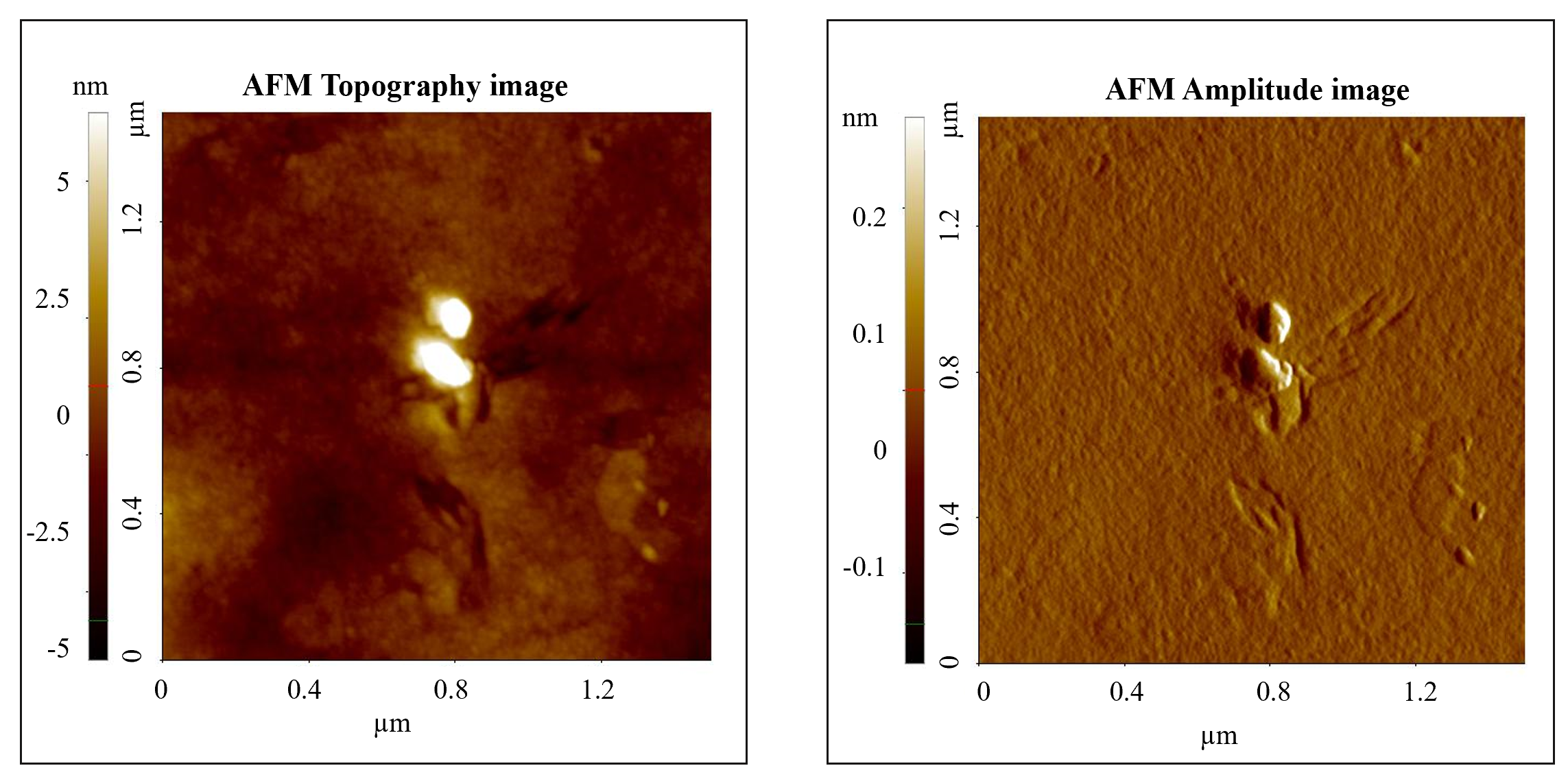


**Supplementary Figure 1.** Two-dimensional topography and amplitude AFM images of fixed mitochondria attached to mica.

**Supplementary Figure 2.** Normalized displacement obtained by the optical nanomotion technique of untreated, PFA-fixed, rotenone-exposed and GA-fixed mitochondria.

**Supplementary Figure 3.** Optical nanomotion displacement of mitochondria in mitochondria storage buffer (control) and buffer supplemented with DMSO (DMSO). p > 0.05 (p= 0.1237), no significant differences were found.

**Supplementary Figure 4.** Optical nanomotion displacements of isolated mitochondria, *E. coli* and *C. albicans*.
